# Estimating travel reduction associated with the use of telemedicine by patients and healthcare professionals: proposal for quantitative synthesis in a systematic review

**DOI:** 10.1186/1472-6963-11-185

**Published:** 2011-08-08

**Authors:** Richard Wootton, Kambiz Bahaadinbeigy, David Hailey

**Affiliations:** 1 Norwegian Centre for Integrated Care and Telemedicine, University Hospital of North Norway, PO Box 6060, N-9038 Tromsø, Norway; 2 Australian e-Health Research Centre, CSIRO, Leeuwin Centre, 65 Brockway Road, Floreat, WA 6014, Australia; 3 School of Information Systems and Technology, University of Wollongong, Wollongong, NSW 2522, Australia; 4 Research Centre for Modelling in Health, Kerman University of Medical Sciences, Kerman, Iran

## Abstract

**Background:**

A major benefit offered by telemedicine is the avoidance of travel, by patients, their carers and health care professionals. Unfortunately, there is very little published information about the extent of avoided travel. We propose to undertake a systematic review of literature which reports credible data on the reductions in travel associated with the use of telemedicine.

**Method:**

The conventional approach to quantitative synthesis of the results from multiple studies is to conduct a meta analysis. However, too much heterogeneity exists between available studies to allow a meaningful meta analysis of the avoided travel when telemedicine is used across all possible settings. We propose instead to consider all credible evidence on avoided travel through telemedicine by fitting a linear model which takes into account the relevant factors in the circumstances of the studies performed. We propose the use of stepwise multiple regression to identify which factors are significant.

**Discussion:**

Our proposed approach is illustrated by the example of teledermatology. In a preliminary review of the literature we found 20 studies in which the percentage of avoided travel through telemedicine could be inferred (a total of 5199 patients). The mean percentage avoided travel reported in the 12 store-and-forward studies was 43%. In the 7 real-time studies and in a single study with a hybrid technique, 70% of the patients avoided travel. A simplified model based on the modality of telemedicine employed (i.e. real-time or store and forward) explained 29% of the variance. The use of store and forward teledermatology alone was associated with 43% of avoided travel. The increase in the proportion of patients who avoided travel (25%) when real-time telemedicine was employed was significant (*P *= 0.014). Service planners can use this information to weigh up the costs and benefits of the two approaches.

## Background

Telemedicine can be defined as the use of information and communications technology to provide health care services for persons who are some distance from the provider. Thus it is a technique, or process for service delivery, which makes use of various technologies to exchange information. A major benefit offered by telemedicine is the avoidance of travel, by patients, their carers and health care professionals. Use of telemedicine can reduce the cost and time of any travel required, and lead to faster delivery of medical services. Avoided travel is also an environmental benefit of telemedicine, and one that is becoming increasingly important [1].

In designing a new telemedicine service, the planner needs to know what avoided travel to expect. Clearly this will depend on the types of patients and the specialties involved. It is also likely to depend on the type of telemedicine employed (i.e. real time or store and forward) and a number of other factors. Unfortunately, only limited information exists about the extent of savings that can be expected for different telemedicine applications, and there is very little published information about avoided travel. Relatively few publications have provided details of travel savings through routine use of established telemedicine applications in health care systems.

We therefore propose to undertake a systematic review of literature which reports credible data on the reductions in travel associated with the use of telemedicine. The intention is to summarise the information about the proportion of avoidable travel possible through use of different telemedicine applications in different contexts. For the purposes of our study avoidable travel is defined as the proportion of consultations or other episodes of care using telemedicine in which patients, carers or health professionals do not have to travel to another centre.

## Methods/Design

### Review

The review will identify studies in which the percentage of avoided travel or avoided referrals through use of telemedicine was measured or inferred. This information will then be used to make the best estimate possible of the true value of the percentage travel avoided through use of telemedicine in different medical specialties. Because telemedicine is often employed in very different ways within a given speciality, it is necessary to take account of the relevant factors. For example, telemedicine has been used in cardiology for many years. However, there are obvious differences between say the use of telecardiology to support general practitioners and the use of telecardiology in paediatric work. In the former, there may be store-and-forward transmission of an ECG recorded in an adult patient suspected of having a myocardial infarction; in the latter, there may be real-time transmission of an echocardiograph from a new born baby suspected of a cardiac defect.

Thus for each "set of circumstances" (i.e. for each telemedicine application in a given specialty), the values that are reported in the reviewed papers, or that can be derived from the data that they include, can be considered as an underlying true value plus an error component:

where Y_i _is the predicted value for avoided travel in a given set of circumstances, i;

β_0 _is the baseline value (intercept);

β_i _is the coefficient relevant to the circumstances, X_i_;

ε_i _is the error term.

The error term may have both systematic and random components. The question of defining the systematic element is complex and not readily addressed [2]. We propose to define the inclusion/exclusion criteria for reported studies so that the mean systematic errors associated with avoided travel can be ignored for the purposes of our review.

**Figure 1:**
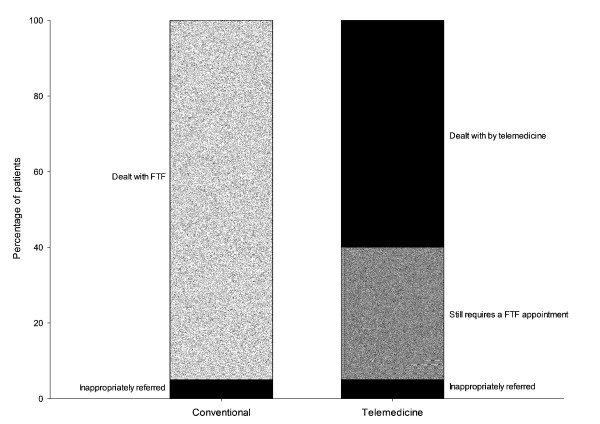
**Example of avoided travel**. The data represent the results of a hypothetical study in which 100 patients were seen by a dermatologist in hospital, and 100 patients were seen by a dermatologist using telemedicine. In both groups, 5% of the patients were considered to have been referred inappropriately (e.g. because the underlying skin problem had resolved by the time they were seen by the specialist). In the telemedicine group, 60 of the patients were managed successfully by telemedicine but 35 still required a conventional appointment. The effect of telemedicine could be considered as avoiding travel for 60% of those in the telemedicine group, although it could also be argued that the proportion was slightly higher (60 out of 95 appropriate referrals).

Our original aim was to find studies where the effect of using telemedicine on patient travel was explicitly addressed, e.g. comparative studies in which patient travel was reported for a telemedicine arm and for a control arm. However, a preliminary review showed that there were very few studies where this had been done. Therefore we intend widening the scope to include non-comparative studies which report the percentage of patients in a telemedicine group who avoid travel. The subtle difference here is that we know that in any usual care group, a proportion of such patients *could *avoid travel. That is, in some patients who present at an outpatient clinic, for example, in the ordinary way, there will be some who will have been referred inappropriately, see Figure 1. To reduce the risk of bias in the selected studies influencing our estimates of avoided travel, we will assess reliability of data, study performance and sample size. Scores from our assessments of these factors will be used to calculate a weighting factor.

### Literature search strategy

Computerized literature searches will be performed using MEDLINE, HealthSTAR, EMBASE, CINAHL and the Cochrane Database of Systematic Reviews, with no date restrictions. The search strategy will make use of both controlled vocabulary, such as the National Library of Medicine's MeSH (Medical Subject Headings), and appropriate keywords.

Two approaches are possible. In the first, the search terms focus on the concepts of travel savings or avoided referrals through the use of telemedicine generally:

(phrase A) **AND **(phrase B) **AND **(phrase C)

where

phrase A = Telemedicine OR Telehealth OR telecare OR teleconsultation

phrase B = Avoid* OR prevent* OR decrease OR reduce OR unnecessar* OR sav* OR prevent

phrase C = Travel OR referral OR visit OR admission OR hospitalization OR transfer* OR transport* OR cost saving* OR appointment OR cost stud* OR remote consultation* OR economic*

In the second, the search terms focus on telemedicine and its use in specific specialties:

(phrase A) **AND **(phrase D)

where

phrase D = Dermatology (for example)

In addition, telemedicine journals will be hand searched to identify any further relevant publications.

Grey literature (i.e. literature that is not commercially published) will be identified by searching the websites of health technology assessment and related agencies, and professional associations.

### Selection criteria

The inclusion criteria will be:

(1) publications that consider travel or travel-related issues for patients, carers or health professionals, and include appropriate details on the data, methods of analysis and outcomes applicable to avoidance of travel. The proportion of avoided travel will be reported directly, or be easily calculable using the information in the paper;

(2) publications reporting studies in which at least 15 patients were managed using telemedicine;

(3) publications that contain an electronic abstract;

(4) publications in the English language.

The exclusion criteria will be:

(1) any paper not reporting the sample size or methodology for calculating the percentage of avoided travel;

(2) articles where only anecdotal information on travel-related issues is given, without credible data and analysis;

(3) single case studies and series with fewer than 15 individuals;

(4) duplicate publications.

### Article selection and data extraction

At least two reviewers will independently apply the selection criteria to the titles and abstracts returned by the literature search. Full-text articles will be obtained for abstracts that meet the selection criteria and for undecided articles. Articles will be perused and included for review if they meet the selection criteria. Any discrepancies between reviewers' decisions will be resolved by consensus. At least two reviewers will independently extract data from the selected publications using a data abstraction form, created *a priori*. Any disagreements will be resolved by consensus.

Information extracted will include the medical specialty; setting and duration of study; patient numbers and characteristics; details of data related to avoidance of travel through use of telemedicine; telemedicine modality.

### Assessment of risk of bias

Each study will be assigned to one of three classes related to the reliability of data:

(1) studies in which data on avoidance of travel or referrals through use of telemedicine were collected prospectively (score 3);

(2) prospective studies in which estimates of travel or referrals avoided through use of telemedicine were based on the opinions of the investigators (score 2);

(3) retrospective or hypothetical studies (i.e. "thought" experiments), including appraisal of patient records (score 1).

These scores reflect differences in strength of evidence between prospective and retrospective studies, and between prospective collection of data and estimates based on expert opinion.

In assessing *study performance*, four areas will be considered:

(1) subjects: how recruited to the study;

(2) intervention: adequate description of the intervention used for the treatment of patients;

(3) data analysis: details of how the data were collected and analysed;

(4) outcomes: inclusion of individual subject data or a statistical summary, details of missing results, drop outs.

For each of these items, there will be an expectation of reasonable quality data and clear presentation of details. If all these areas are adequately covered, study performance can be regarded as good quality (score 3). If there are limitations or omissions in one of the items, the study performance is fair (score 2). Limitations in more than one item would indicate poor performance quality (score 1).

The *sample size *will be taken as the number in the telemedicine intervention group. The sample size will be categorized as:

(1) large, i.e. ≥150 (score 3);

(2) medium, i.e. 50-149, (score 2);

(3) small, i.e. 15-49 (score 1).

For the purposes of our analysis each of the indices (study design, study performance and sample size) will be regarded as equally important. For each study, the scores for each index (3, 2 or 1) will be summed to give an individual study *weighting factor *with a minimum of 3 and a maximum of 9 (i.e. a 7-point scale).

At least two reviewers will determine scores for study design, study performance and sample size for each study. Any disagreements will be resolved by consensus.

### Data synthesis

We expect that the number of publications dealing explicitly with travel savings through use of telemedicine will be small. We therefore propose a methodology that will take into account all the credible evidence, even some that is weak, rather than attempting to analyse effect size on a restricted dataset of the more rigorous studies.

The conventional approach to quantitative synthesis of the results from multiple studies is to conduct a meta-analysis. However, there are well known difficulties about attempting to combine results from studies which are too dissimilar [3]. Our hypothesis is that far too much heterogeneity exists between available studies to allow a meaningful meta-analysis of the data on avoided travel when telemedicine is used across all possible settings. There is also a dearth of randomized controlled trials in telemedicine. It follows that a different approach is required.

We propose instead to consider all credible evidence on avoided travel through telemedicine, and to fit a linear model which takes into account the relevant factors in the circumstances of the studies performed. We propose the use of stepwise multiple regression to identify which factors are significant. Possible factors are listed in Table 1.

**Table 1:** Factors which may influence the avoided travel resulting from a particular telemedicine application

Modality	Store and forward
	Hybrid
	Real time
	
Setting - referral source	Home
	Primary care
	Hospital
	
Setting - referral location	Metropolitan
	Rural
	
Travel distance or travel time	Long (> = 1 hour)
	Short (< 1 hour)
	
Patient age	Young (fetal and paediatric)
	Adult
	Elderly
	
Urgency	Elective
	Emergency
	
Purpose	Diagnosis
	Management (treatment, monitoring)
	
Health system	Private
	Public
	
Specialty	Cardiology
	Dermatology
	Psychiatry
	etc (see Table 4)

In accordance with the MOOSE recommendations for reporting the results of meta-analyses of observational studies, we will include graphical summaries of study estimates and any combined estimate, a table listing descriptive information for each study, results of sensitivity testing and any subgroup analysis, and an indication of the statistical uncertainty of findings [4].

The process of data synthesis is thus:

(1) study selection following the literature search;

(2) data extraction, including calculation of study weighting factors;

(3) regression analysis.

## Example

**Figure 2:**
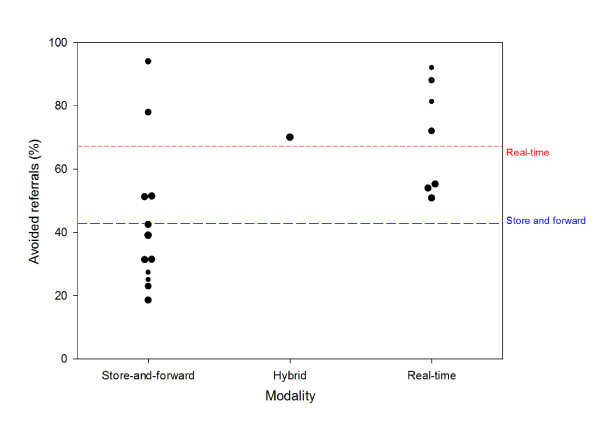
**Avoided referrals from primary care**. Avoided referrals as a result of the use of teledermatology in referrals from primary care (20 studies from 1997-2010). The areas of the symbols are proportional to the study weights. The broken lines represent the avoided travel expected from the fitted model (see text).

The methodology above is illustrated by the example of teledermatology. A preliminary review of the literature (April 2010) found 20 studies in which the percentage of avoided travel through telemedicine could be inferred (see Table 2). The total number of patients in these studies was 5199. The mean percentage avoided travel in the 12 store-and-forward studies was 43%. The mean proportion of patients who avoided travel in the 7 real-time studies and in a single study using a hybrid technique was 70%, see Figure 2.

**Table 2:** Data for 20 teledermatology studies in which travel avoided was estimated

Author	Year	Percentage of patients who avoided travel	Study design (a)	Sample size	Weight (b)	Country	Modality (c)	Urgency	Referral setting	Patient age	Comment
Whited J [10]	2002	18.5	3	135	8	USA	S/F	Elective	Primary care	Mean 61 years (SD 13.8)	
Bowns I [11]	2006	42.4	3	92	8	UK	S/F	Elective	Primary care	16 years and over	Excludes children
White H [12]	1999	25.0	2	40	4	UK	S/F	Elective	Primary care	Not stated	
Loane M [13]	2000	31.3	3	96	8	UK	S/F	Elective	Primary care	7 months to 81 years	Loane 2000 is two arms of the same study
Taylor P [14]	2001	31.4	2	376	8	UK	S/F	Elective	Hospital OPD clinic	Not stated	376 tele-assessments; 194 patients
Knol A [15]	2006	51.4	2	306	8	Netherlands	S/F	Elective	Primary care	0-96 years (in larger sample of 505 patients)	
Wootton R [16]	2000	53.9	3	102	8	UK	R/T	Elective	Primary care	4 months to 89 years (in larger sample of 204 patients)	
Loane M [13]	2000	55.2	3	96	8	UK	R/T	Elective	Primary care	7 months to 81 years	Loane 2000 is two arms of the same study
Lamminen H [17]	2000	72.0	2	25	7	Finland	R/T	Elective	Primary care	Mean 45 years (range 4-92)	
Granlund H [18]	2003	81.3	1	16	4	Finland	R/T	Elective	Primary care	Mean 40 years (SD 21), both groups	70% of 23 patients in video group said they had not visited hospital at 6-month follow-up
Chen T [19]	2010	94.0	1	429	6	USA	S/F	Elective	Primary care	Mean 5.9 years (range 0-12 years 11 months)	Only children (12 years or younger)
Romero G [5]	2009	70.0	3	368	9	Spain	Hybrid	Elective	Primary care	Mean 36 years (range 2 months -86 years) [but this includes an extra control group]	192 pts had S/F alone; 176 had S/F and then Real-time
Eminovic N [20]	2009	39.0	3	200	9	Netherlands	S/F	Elective	Primary care	Mean approx 43 years	
Moreno-Ramirez D [21]	2007	51.2	2	2009	8	Spain	S/F	?Urgent	Primary care	Mean 41.5 years	Pigmented skin lesions -- possible skin cancer
Klaz I [22]	2005	77.9	2	435	7	Israel	S/F	Elective	Primary care	Mean 22.4 years (range 18-39)	Excludes children
Eminovic N [23]	2003	22.9	2	96	7	Netherlands	S/F	Elective	Primary care	Mean 35 years	Patients provided the images
Oakley A [24]	2000	88.0	2	109	6	New Zealand	R/T	Elective	Primary care	Mean 41 years (range 1 month to 94 years) [may include non-telederm patients as well)	
Burgiss S [25]	1997	92.0	1	87	4	USA	R/T	Elective	Primary care	Not stated	
Gilmour E [26]	1998	50.8	3	61	8	UK	R/T	Elective	Primary care	3 months to 83 years (for all 126 patients)	
Jemec G [27]	2008	27.3	1	121	4	Denmark (Faroes)	S/F	Elective	Primary care	Mean 37 years (SD 20)	

(a) 1 = prospective; 2 = estimated; 3 = retrospective (see text)

(b) score 3-9 (see text)

(c) S/F, store-and-forward; R/T, real-time

Dummy coding can be used to represent putative predictive variables as follows:

Modality - baseline (store and forward); hybrid; real-time

Country - baseline (North America); Europe; Rest of world

Patient group - baseline (all ages); only adults; only children

A linear model with all three putative predictors fits the data well (R^2 ^= 0.761). However, the coefficients for country and for patient group are not significant. Simplifying the model to include only the significant predictor Modality produces R^2 ^= 0.292. It also shows that the coefficients for Hybrid and for Real-time are almost identical in size. If the single study in which a hybrid technique was used [5] is reclassified as real-time, then the model (R^2 ^= 0.291) is:

where A is percentage of avoided travel;

M = 0 if store-and-forward is used and M = 1 if real-time telemedicine is used.

That is, use of store and forward teledermatology was associated with avoided travel for a mean of 43% of the patients in the studies (*P *< 0.001). The improvement in the proportion of patients who avoided travel (25%) when real-time telemedicine was employed is significant (*P *= 0.014), Table 3.

**Table 3:** Coefficients of fitted model (using Modality as a predictor)

	Unstandardized coefficients		Standardized coefficient		
	B	SE	Beta	t	*P*-value
(Baseline)	42.816	5.616		7.6	< 0.001
Dummy_modality2	24.487	9.011	0.539	2.7	0.014

## Discussion

Our proposed review is intended as an appraisal of the travel savings achievable through telemedicine. Our methodology takes a relatively simple approach to the assessment of studies, and makes a number of assumptions. For example, the decision to allocate equal weights to reliability of data, study performance and sample size is arbitrary. An earlier approach to quality assessment of telemedicine studies used differential weights, with the weight for study performance being twice that for study design [6]. It would also be possible to classify both reliability of data and study performance in more detail [6,7]. These are matters that might be considered in future studies.

The use of stepwise regression to identify significant factors has been used previously in meta-regression [8,9]. Because of the relatively small dataset in the dermatology example (n = 20), it was not possible to explore the significance of all possible factors. However, the model appeared to produce sensible results when three factors were investigated. With a large dataset, such as that likely to result from the proposed systematic review, it would be possible to explore most or all of the possible factors in Table 1 and to do so using a robust exploration technique, such as resampling.

Some matters may need to be developed further after the review commences. One of these is how the studies will be classified in terms of the medical specialties involved, since there does not appear to be an internationally-accepted system for classification. Our proposal, a pragmatic scheme, is shown in Table 4. The significance of other factors, such as study setting and urgency of consultations is unclear at this stage and will need to be explored after more data become available.

**Table 4:** Categories of specialties

Specialty	Subspecialty	... Specialty	... Subspecialty
Allied Health		Pathology	
	Chiropractice		Anatomical
	Occupational therapy		Bacteriology
	Physiotherapy		Breast cancer
	Podiatry		Clinical chemistry
	Rehabilitation		Cytopathology
	Speech therapy		Dermatology
Anaesthesia			Forensic
	Intensive care		Haematology
	Obstetric		Histology
	Pain		Immunology
Emergency medicine			Microbiology
			Neuropathology
General practice		Public Health	
			Environmental
Internal Medicine			Occupational
	Cardiology		Preventive
	Dermatology	Radiology	
	Endocrinology		Diagnostic
	Gastroenterology		Neuroradiology
	Genetics		Nuclear medicine
	Geriatrics		Oncology
	Haematology		Radiography
	Hepatology		Radiotherapy
	Immunology		Ultrasound
	Infectious diseases	Surgery	
	Intensive care		Abdominal
	Maternal/fetal		Burns
	Neurology		Colon/Rectal
	Oncology		ENT
	Ophthalmology		Hand
	Preventive		Head and neck
	Renal		Maxillo-Facial
	Reproductive		Neurosurgery
	Respiratory		Oncology
	Rheumatology		Ophthalmology
	Sexual and reproductive health		Orthopaedics
	Thoracic		Plastic
	Tropical diseases		Spinal
	Tropical medicine		Thoracic
Mental Health			Urology
	Psychiatry		Vascular
	Psychology		
Nursing			
	Diabetes and pregnancy		
	Midwife		
	Paediatrics		
Obstetrics and gynaecology			
	Colposcopy		
	Oncology		
	Reproductive		
	Urogynaecology		
Other			
	Dietetics		
	Hospital pharmacy		
	Proxy consultant		
	Wound care		
Paediatrics			
	Cardiology		
	Dentistry		
	Dermatology		
	Endocrinology		
	Gastroenterology		
	Genetics		
	Haematology		
	Immunology		
	Infectious diseases		
	Intensive care		
	Metabolic diseases		
	Neonatal		
	Neurology		
	Oncology		
	Physiotherapy		
	Psychiatry		
	Radiology		
	Renal		
	Respiratory		
	Rheumatology		
	Surgery		

Numbers of studies in some specialties may be small and will therefore provide estimates of travel savings which have a high level of uncertainty. Nevertheless, such results are expected to provide helpful initial indications and should assist in defining areas for future work.

The example analysis of dermatology studies suggests that substantial avoided patient travel can be expected through the use of store-and-forward telemedicine. The results also provide an estimate of the additional value of real-time telemedicine, a technique which is generally speaking less convenient and more expensive than store-and-forward telemedicine. Service planners can use this information to weigh up the costs and benefits of the two approaches. A systematic review across the whole of telemedicine with data synthesis in accordance with the proposals in the present paper therefore appears likely to provide important information for those planning the future introduction of telemedicine services around the world.

## Competing interests

The authors declare that they have no competing interests.

## Authors' contributions

KB was responsible for the research question. RW formulated and developed the methodological concept. All authors contributed to reviewing data and writing the manuscript. All authors read and approved the final manuscript.

## Pre-publication history

The pre-publication history for this paper can be accessed here:

http://www.biomedcentral.com/1472-6963/11/185/prepub
